# Comparison of Dye Adsorption of Chitosan and Polyethylenimine Modified Bentonite Clays: Optimization, Isotherm, and Kinetic Studies

**DOI:** 10.1021/acsomega.3c07509

**Published:** 2024-02-14

**Authors:** Nilay Kahya, Begüm Şen, Demet Berber, Nevin Öztekin

**Affiliations:** Department of Chemistry, Istanbul Technical University, Maslak, Istanbul 34469, Turkey

## Abstract

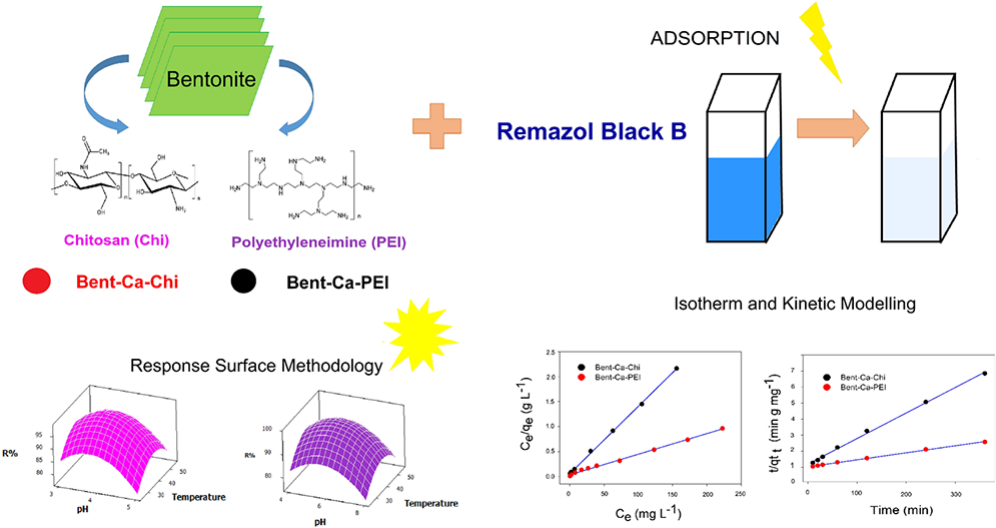

The aim of this study was to compare the effect of modifying calcium bentonite (Bent-Ca) clay with two cationic polymers, chitosan (Chi) and polyethylenimine (PEI), on the removal of remazol black B (RB-B) dye from an aqueous solution. The samples were characterized by using scanning electron microscopy, X-ray diffraction, and Fourier-transform infrared spectroscopy. The fractional factorial design of 2^(6–1)^ was applied to investigate the effects of pH, temperature, amount of adsorbent, initial dye concentration, contact time, and shaking rate on the adsorption process. To further optimize RB-B removal from an aqueous solution, a Box–Behnken design with three factors and a response surface methodology was used. The optimum conditions were a pH of 3.77, a temperature of 40.45 °C, and an initial RB-B concentration of 77.27 mg L^–1^ for Bent-Ca-Chi, whereas for Bent-Ca-PEI, the optimum conditions were a pH of 5.53, a temperature of 41.06 °C, and an initial dye concentration of 238.89 mg L^–1^. To understand the adsorption behavior, the Langmuir and Freundlich isotherms were fitted to the experimental data. It was found that the Langmuir isotherm model matched well with the dye adsorption by Bent-Ca-Chi and Bent-Ca-PEI. The kinetics study was performed using three kinetic models: pseudo-first-order, pseudo-second-order, and intraparticle diffusion models. Among these models, the RB-B dye kinetics were best represented by the pseudo-second-order model equation for the adsorbents.

## 1 Introduction

Dye pollution resulting from the residuals of many types of synthetic dyes from industries, such as textile, printing, and tannery industries, has become a significant environmental problem due to uncontrolled wastewater production. Residuals of synthetic textile dyes have led to pollution of many aquatic systems as a result of unplanned waste disposal. The existence of polluting dyes in wastewater adversely affects aquatic organisms^1−3^ and can also pose health risks to humans, causing cancer and skin-related diseases.^4−6^ Various physical, oxidative, and biological methods have been proposed for the removal of textile dye from aquatic systems.^7−9^ Among these methods, adsorption is a standard and straightforward process for dye removal. Previous studies have demonstrated the successful use of this technique for the removal of several dyes, such as basic yellow and malachite green,^10^ methylene blue,^11−13^ crystal violet,^14,15^ navy blue,^16^ congo red,^17^ and remazol black B (RB-B).^18^ RB-B, also known as reactive black 5, is a member of the azo dye family and is commonly used as a colorant in the textile industry. This dye was chosen in this study to investigate dye removal using an adsorption technique.

Clays are widely used adsorbents for dye removal studies and are most effective in their raw or modified structures.^19−22^ Bentonite (Bent) is an abundant, low-cost clay. However, its application for anionic dyes is limited due to its negatively charged surface; therefore, surface modifications are needed so that the clay can be used as an adsorbent.^23^ Amended Bent can be obtained from nonionic, cationic, and anionic polymers according to the different charges of the polymer modifiers.^24^

Chitosan (Chi) is a polysaccharide produced by the deacetylation of chitin in basic media. Chitin is the skeletal material of many crustaceans and the second most abundant polysaccharide on Earth after cellulose. Chi has a positive charge at a pH of 6.5 and is reported to be a suitable adsorbent, particularly for reactive azo and organic dyes.^25−27^ Polyethylenimine (PEI) is a high positive charge density polymer that has been utilized in dye adsorption studies thanks to its cationic character.^28−30^ Chi and PEI, as cationic charged polymers, have been used as cationic charge enhancers to increase the adsorption capacity of Bent clay for one anionically charged surfactant or two anionically charged dyes.^31−33^ These polymers are applied to modify Bent to prepare a clay-based adsorbent with improved dye removal capacity through an enhanced interaction with the dyes. Abukhadra et al. prepared a Chi-cobalt oxide altered Bent composite adsorbent for anionic azo dyes, like congo red, and it successfully adsorbed the dye onto the Bent-based clay.^34^ Du et al. studied the removal of the anionic amino black 10 B dye using a PEI/trimethoxysilane-modified Bent adsorbent.^35^

By employing a simple and previously established method,^36^ Bent-Ca clay was modified using cationic charged polymers, namely Chi and PEI, without using any toxic solvent in the process. The prepared clay adsorbents were evaluated for their capability to remove the selected model dye RB-B in an aqueous medium, and the dye removal capabilities of the adsorbents were then compared. A fractional factorial design (FFD) was used to determine the factors affecting RB-B adsorption. Based on the FFD results, response surface methodology combined with the Box–Behnken design (BBD) was applied to understand the interaction between independent variables and to optimize the RB-B adsorption process by clay adsorbents with Bent skeletons restructured with Chi or PEI. Furthermore, the isotherms and kinetics of the dye-adsorption process were investigated using these novel adsorbents. The clay samples were also characterized by scanning electron microscopy (SEM), X-ray diffraction (XRD), and Fourier-transform infrared (FTIR) analyses to investigate the morphological and structural changes resulting from the polymer modification of Bent-Ca.

## 2 Experimental Section

### 2.1 Materials

Natural Bent-Ca clay samples were collected from the Enez region, located near the border between Greece and Turkey. Chi (low molecular weight, degree of deacetylation of 75–85%, and viscosity of 20–300 cP), PEI (molecular mass range of 6 × 10^5^ – 1 × 10^6^ g/mol), and boric acid were purchased from Fluka (Buchs, Switzerland). Remazol black B (dye content ≥50%) was purchased from Sigma-Aldrich (St. Louis, MO, USA). Acetic acid (glacial ≥99.9%) was obtained from Merck (Darmstadt, Germany). *O*-phosphoric acid (85%) was purchased from Riedel-de Han (Seelze, Germany). All of the solutions were prepared using deionized water obtained from the Elga Purelab Option-Q system.

In this study, Britton–Robinson (BR) aqueous universal buffer solutions with pH values between 2 and 8 were prepared by mixing appropriate volumes of acidic and basic buffer components. The acidic buffer component comprises 0.4 M *o*-phosphoric acid, 0.4 M boric acid, and 0.4 M acetic acid in a 250 mL solution. Buffer solutions were used to adjust the pH of the dye solutions in adsorption vessels.

The adsorption vessels were shaken in a Nüve ST-402 shaking water bath (Ankara, Turkey). A Nüve NF 1200 centrifuge (Ankara, Turkey) was used to centrifuge the adsorbed dye solutions. Spectrophotometric measurements of RB-B dye solutions were performed using a Shimadzu UV-1800 spectrophotometer (Kyoto, Japan). pH measurements were performed by using an Orion Dual Star pH-ISE meter (Thermo Fisher Scientific, Waltham, MA, USA) equipped with a combined glass pH electrode. SEM measurements of the clay samples were performed by using a Tescan Vega3 scanning electron microscope (TESCAN, Brno, Czech Republic). Prior to the SEM measurements, the clay samples were thinly sputtered with gold. XRD analysis of the modified clay samples was performed using a Bruker D8 Advance Series diffractometer (Billerica, MA, USA). FTIR data were obtained using a PerkinElmer 102 Spectrum One FTIR spectrometer (Shelton, CT, USA).

### 2.2 Preparation of Adsorbents

The amount of polymer adsorbed on Bent clay was determined based on our previous study, which reported the adsorption capacity of PEI onto Bent clay to be 145 mg/g clay.^36^ In this study, this ratio was nearly quintupled as the polymer was adsorbed onto neat calcium Bent. For the adsorption process, 5 g of Bent clay was dispersed in 100 mL of distilled water and stirred continuously for 24 h. PEI and Chi were added to the Bent suspensions at a concentration of 800 mg of polymer per gram of clay from their preprepared stock solutions, which were prepared in water and 1% (v/v) acetic acid solution, respectively. The PEI-clay and Chi-clay mixtures were shaken at 250 rpm and 25 °C for 24 h. Then, the clay suspensions were centrifuged at 5000 rpm for 30 min to obtain a solid product. Excess PEI and Chi were removed by washing the precipitate with distilled water. The wet clay samples adsorbed with PEI and Chi were dried in an oven at 55 °C for 48 h. The resulting modified clay samples were named Bent-Ca-PEI and Bent-Ca-Chi, respectively. The solid clay samples were collected and passed through a 90 μm sieve. In their dry states, Bent-Ca-PEI and Bent-Ca-Chi adsorbents were used in RB-B dye adsorption studies.

### 2.3 Adsorption Experiments: Optimization, Isotherm, and Kinetic Studies

The adsorption experiments for FFD and BBD were performed in stoppered plastic vessels containing 10 mL of the dye solution. Before each experiment, the RB-B dye solution was taken from a freshly prepared stock solution at 5000 ppm. The dye solution was then diluted with deionized water to the desired initial concentration, and the buffer solution was added to the vessels to stabilize the pH. The adsorbent clay samples were added to the adsorption vessels, which were shaken in a temperature-controlled water bath. At the end of the adsorption process, the dye solutions were placed in centrifuge tubes and centrifuged at 5000 rpm for 30 min to precipitate the dye-adsorbed clay from the supernatant. The amount of RB-B remaining after adsorption was calculated from the calibration curve by measuring the absorbance of the supernatant dye solution at 595 nm by using a UV–vis spectrophotometer. The amount of RB-B adsorbed at equilibrium (*q*_e_) was determined as follows:
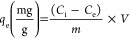
1where *C*_i_ and *C*_e_ are the dye concentrations (mg L^–1^) before and after adsorption, respectively. *V* is the volume of the dye solution (L), and *m* is the weight of the adsorbent (g). The RB-B removal percentage was calculated using the following equation:
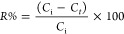
2where *C*_i_ and *C*_*t*_ are RB-B concentrations (mg L^–1^) initially and at time *t*, respectively.

Dye adsorption for isotherm modeling was performed at an initial RB-B concentration of 5–300 mg L^–1^. The initial pH values of the adsorbate solutions were adjusted to 4 and 6 for Bent-Ca-Chi and Bent-Ca-PEI, respectively, by the addition of the buffer solution. The final volume of the dye solution was made up of 25 mL in a volumetric flask made of glass. The amounts of Bent-Ca-Chi and Bent-Ca-PEI added to the dye solution in the plastic vessels were 0.010 g. The adsorbate solutions with adsorbents were shaken at 250 rpm and 40 °C for 240 min in a water bath to reach equilibrium. Dye adsorption experiments for kinetic modeling were conducted at an initial dye concentration of 250 mg L^–1^. The pH of the RB-B solution was fixed at 4 and 6 for Bent-Ca-Chi and Bent-Ca-PEI, respectively, using the buffer solution. The final volume of the solution was 25 mL, and 0.010 g of the clay adsorbent was added to the dye solution. The vessels were shaken at 40 °C at a shaking rate of 250 rpm for 12 h to provide equilibrated adsorption. At a predetermined time, 0.2 mL of the adsorbate dye solution was pipetted and added to 3 mL of distilled water for the absorbance measurement at 595 nm. To maintain the final volume of the dye solution at 25 mL, the volume of 0.2 mL was added from 250 mg L^–1^ dye solution to the vessels.

Adsorption experiments were repeated two times, and the results are given as mean and standard deviation.

### 2.4 Regeneration Studies

The adsorption of dye experiments in each cycle was carried out under the optimum conditions, which were obtained by BBD for Bent-Ca-Chi and Bent-Ca-PEI adsorbents. The desorption of the adsorbent clays was performed by treating the freshly used adsorbent in a wet state with 1 M NaOH for 2 h and then washing it two times with a certain amount of distilled water. The adsorbents were dried at 40 °C for 12 h following the filtration step and then utilized in the next cycle of the adsorption experiment. The dye adsorption experiments to test the reusability of the adsorbents were conducted with two parallel experimental sets.

## 3 Results and Discussion

### 3.1 Characterization of the Clay Samples

#### 3.1.1 SEM

**Figure 1:**
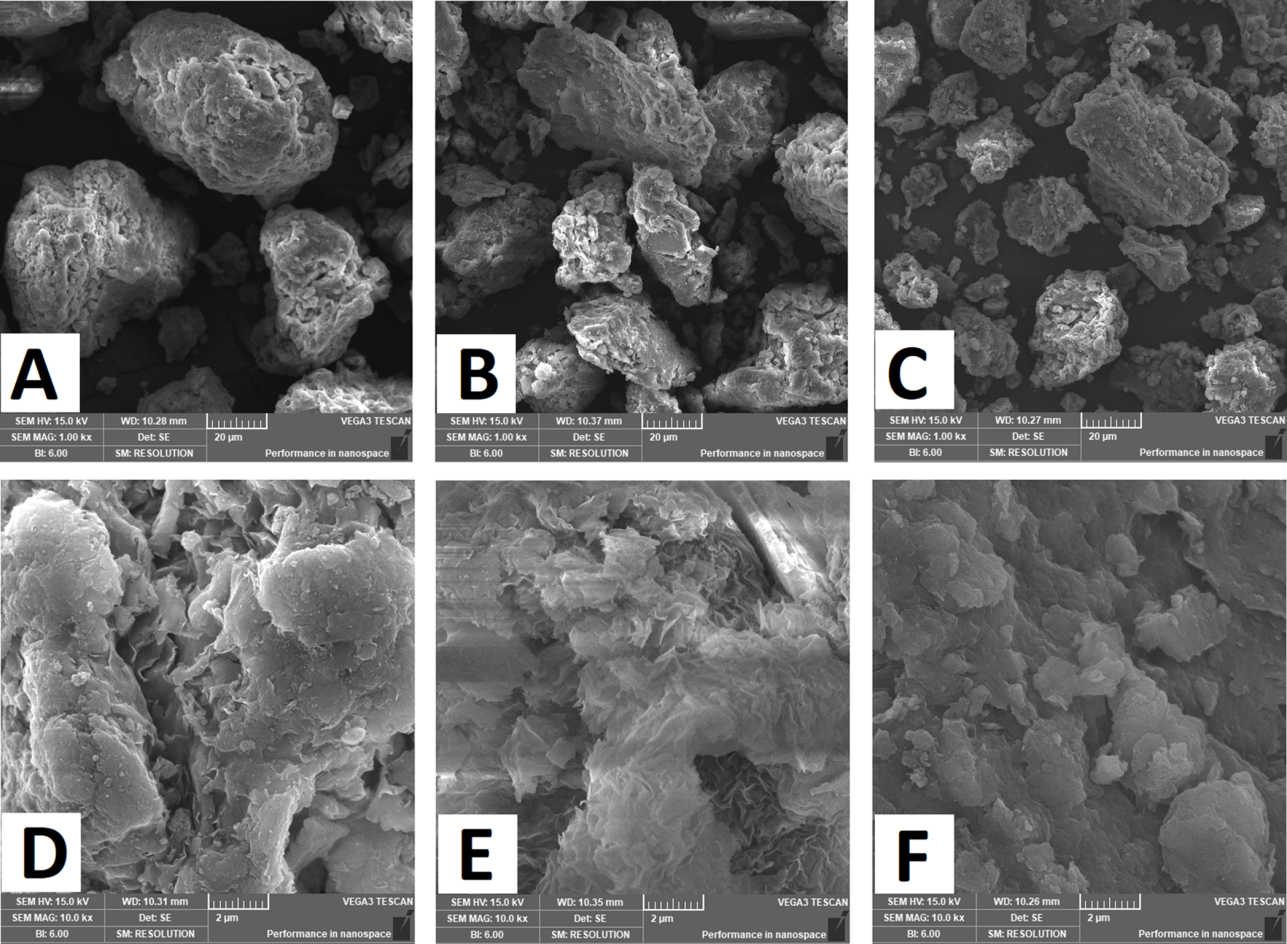
SEM images of Bent-Ca (A, D), Bent-Ca-Chi (B, E), and Bent-Ca-PEI (C, F). Scale bars are 20 and 2 μm at 1000× and 10000× magnifications.

SEM was used to observe the morphological changes in the Bent-Ca clay after modification with Chi and PEI polymers. Figure 1A–F shows the SEM images of the Bent-Ca, Bent-Ca-Chi, and Bent-Ca-PEI samples with scale bars of 20 and 2 μm at magnifications of 1000× and 10 000×, respectively. From Figure 1A–C, it can be observed that the particle size of Bent-Ca significantly decreased owing to the sieving step of the polymer treatment of the clay. As shown in Figure 1D, Bent-Ca exhibits a surface with hollows and embankments that are not regularly formed. The uneven structure of natural Bent has been previously reported in the literature. This uneven structure is consistent with the characteristics of the lamellar and curly surface of raw Bent clay, as previously reported by Liu et al.^37^ Bent-Ca exhibited a distinct thin lamellar structure that consisted of folded flakes. After its interaction with Chi, the exterior surface of Bent evolved into smaller layers of flakes. Nevertheless, the presence of Chi did not completely change the porous structure of the clay, and the lamellar layers of Bent-Ca-Chi are clearly visible in Figure 1E. Although Chi was placed in the layers of clay, the flakes on the surface remained confined in small interspaces. Similarly, da Silva et al. reported that the flaky structure of Na^+^-Bent clay did not significantly change after modification with Chi, with the Chi-Bent nanocomposite retaining the flaked surface from the original Bent structure.^38^ Du et al. showed the irregular lamellar surface of Na^+^-Bent clay, which can be utilized as an adsorbent for an anionic dye after modification with a cationic polymer, poly(2-(acryloyloxy)ethyl)trimethylammonium chloride, before the grafting process.^39^Figure 1F shows that PEI generated a smooth surface morphology of the clay. The globular morphology of Bent-Ca-PEI may be attributed to the coverage of PEI on the Ben-Ca flakes. A study showed that a similar surface variation in Bent modified by (3-glycidyloxypropyl) trimethoxysilane and the PEI composite for the adsorption of amino black 10 B dye results in a smoother morphology compared to the original flaky surface-structured Bent.^35^

#### 3.1.2 XRD

**Figure 2:**
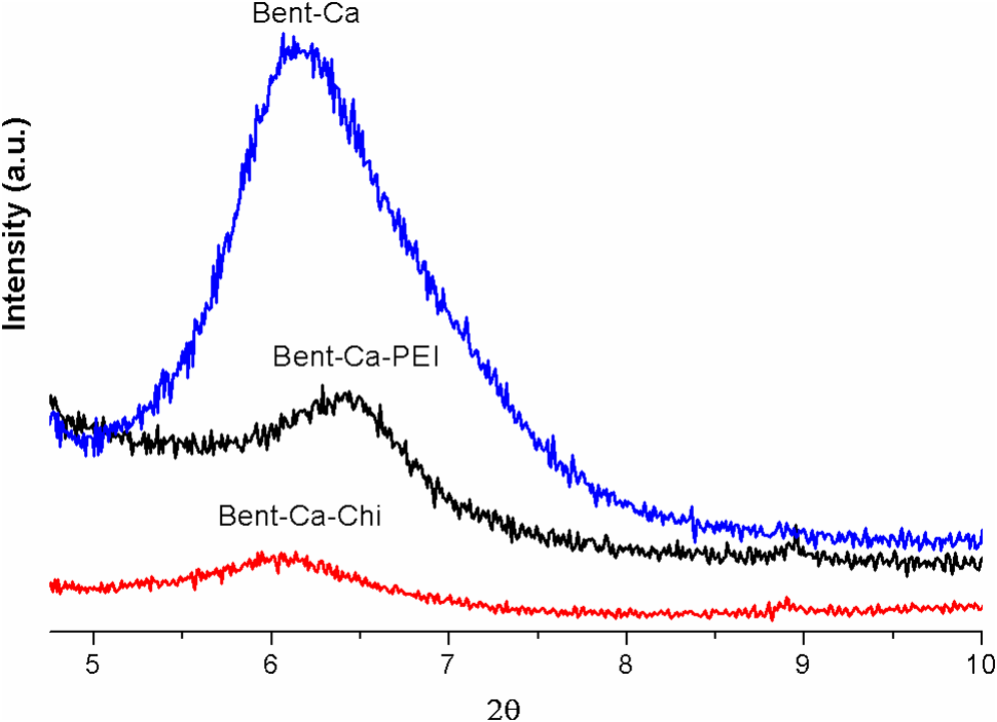
XRD patterns of Bent-Ca, Bent-Ca-Chi, and Bent-Ca-PEI.

To determine the physical characteristics of the clay samples, XRD analysis was performed. The XRD patterns of the unmodified (Bent-Ca) and polymer-modified (Bent-Ca-Chi and Bent-PEI) clays are shown in Figure 2. The diffraction peak at 2θ = 6.23° in the XRD spectra of Bent-Ca indicates that Bent clay possesses a layered structure. Moreover, upon modification of Bent with Chi or PEI polymers, a small shift in the 2θ peak position was observed. The basal spacing (*d*_(001)_) between the clay layers is listed in Table 1. The *d*_(001)_ spacing in Bent-Ca was 1.42 nm. However, after the incorporation of the two different polymers into Bent clay, the *d*_(001)_ values changed to 1.44 nm (Bent-Ca-Chi) and 1.36 nm (Bent-Ca-PEI) for Chi and PEI, respectively. The results showed that Chi led to a 0.02 drift between Bent layers and that spaces exist in the layers enlarged by Chi chains. In contrast to Chi, PEI caused a 0.06 nm decrease in the distance between the Bent clay layers. The reason for this behavior may be attributed to the interaction of PEI with Bent, where the polymer forms hydrogen bonds or undergoes physical interactions with the clay layers. Similar observations have been reported in the literature, where PEI modification of sodium Bent resulted in the confinement of the Bent layers.^40^ The SEM analysis results also confirmed that Chi entered the layers of Bent-Ca, whereas PEI covered the clay’s flakes by interacting with the surface, resulting in a decrease in the interlayer spacing.

**Table 1:** Diffraction Angle (2θ) and the Basal Spacing (*d*_(001)_) of Clay Samples

sample	2θ	*d*_(001)_ nm
Bent-Ca	6.23	1.42
Bent-Ca-Chi	6.14	1.44
Bent-Ca-PEI	6.49	1.36

#### 3.1.3 FTIR

**Figure 3:**
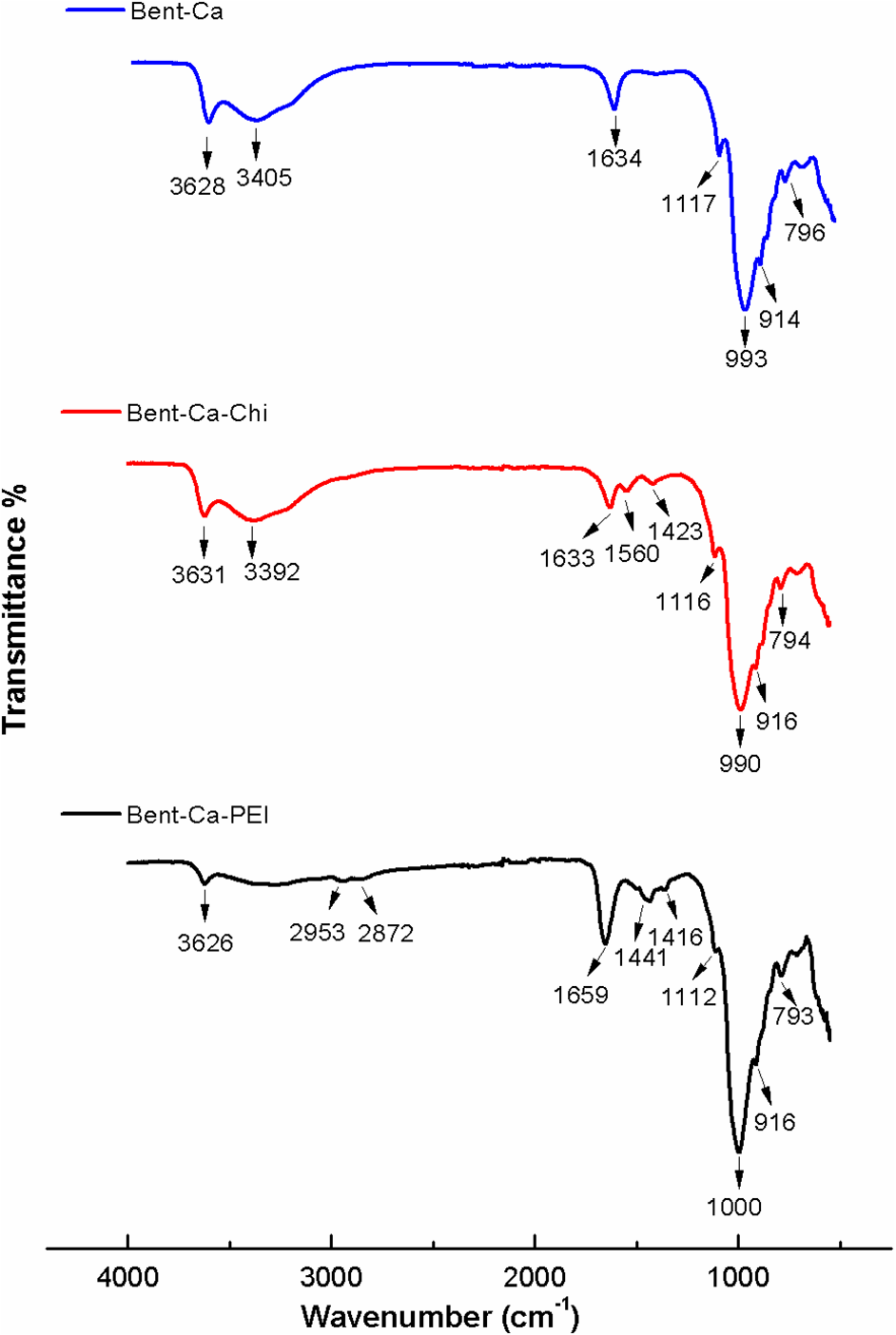
FTIR spectra of Bent-Ca, Bent-Ca-Chi, and Bent-Ca-PEI.

The FTIR spectra of the clay samples are displayed in Figure 3. All clay samples show a peak at ∼3600 cm^–1^, which belongs to the O–H stretching of Bent and arises from the Si–OH group. In the Bent-Ca spectrum, the peak at 3405 cm^–1^ is attributed to the stretching vibration of the absorbed water (H–O–H). The Bent-Ca-Chi clay sample shows a peak at 3392 cm^–1^, resulting from the overlap of the O–H and N–H stretching bands of Chi. The peaks located at 2872 and 2953 cm^–1^ in the Bent-Ca-Chi spectra are associated with symmetric and asymmetric C–H stretching vibrations, respectively. The peak at 1634 cm^–1^ represents the H–O–H bending vibrations of Bent and is observed in the spectra of Bent-Ca and Bent-Ca-Chi. A sharp peak is observed at 1659 cm^–1^ in the Bent-Ca-PEI spectrum, which is associated with the bending vibration of the N–H of PEI. The characteristic Chi peaks appear at 1633 and 1560 cm^–1^, corresponding to the C=O stretching of amide I and the N–H bending of amide II groups. In Bent-Ca, Bent-Ca-Chi, and Bent-Ca-PEI clay spectra, prominent bands are observed at ∼993 cm^–1^ and ∼915 cm^–1^, corresponding to the stretching vibration of Si–O and the bending vibrations of Al–O.^34,35^

### 3.2 Screening Design

Adsorption may be affected by various factors, such as pH, adsorbent amount, and time. As the number of factors of interest increases, it becomes advantageous to use fractional versions of the factorial design. A 2^(6–1)^ FFD was chosen to determine the role and importance of six selected factors (pH, temperature, amount of adsorbent, initial dye concentration, contact time, and shaking rate) in RB-B adsorption. Table 2 presents the low (−1) and high (+1) levels of the factors and their codes. The constructed two-level FFD matrix generated a total of 32 experimental runs of possible combinations of the six factors. Each run was performed two times. The response variable chosen for the experiments’ design was the percentage of RB-B removal (*R*%). The analysis of the results was performed using Minitab statistical software (version 16.0, Minitab Inc., State College, PA, USA). The analysis of variance (ANOVA) test showed that the FFD model was consistent with the experimental results of *R*%, with high *R*^2^ values of 99.48 and 97.46% for Bent-Ca-Chi and Bent-Ca-PEI, respectively. By analyzing the ANOVA test of the standardized effects, it was revealed that pH (A), temperature (B), and initial dye concentration (D) have a significant correlation with *R*% at a 95% confidence level (*p* < 0.05) for both adsorbents. These three factors were identified as the significant variables influencing RB-B adsorption and were subsequently selected as independent variables for further analysis using the BBD.

**Table 2:** Variables, Levels, and Symbols for FFD 2^(6–1)^ Design

	levels		
variables	symbol of effect	low (−1)	high (+1)
pH	A	3	8
temperature (°C)	B	25	55
amount of adsorbent (g)	C	0.01	0.035
initial dye concentration (mg L^–1^)	D	100	350
contact time (min)	E	30	240
shaking rate (rpm)	F	200	500

### 3.3 Optimization of RB-B Adsorption by BBD Combined with RSM

In this study, a BBD with three factors at three levels was employed to determine the effects of these variables on RB-B adsorption. The independent variables selected for the BBD, which were found by FFD, were pH, temperature, and initial dye concentration, which were coded as *X*_1_, *X*_2_, and *X*_3_, respectively. The response variable chosen was the dye-removal percentage (*R*%), which is represented as *Y*. For the independent factors, the BBD-coded levels, which were low (−1), middle (0), and high (+1), are presented in Table 3. The BBD matrix with the coded factors and their levels is presented in Table 4. A total of 15 experiments were conducted to evaluate the effects of significant independent parameters on the RB-B removal percentage. Each experiment was performed twice. According to the response surface methodology, the most common second-order polynomial equation developed to fit the experimental data can be written as eq 3:
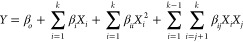
3where β_*o*_, β_*i*_, β_*ii*_, and β_*ij*_ are the constant, linear, quadratic, and interaction coefficients, respectively. The independent variables are represented as *X*_*i*_ and *X*_*j*_, and *Y* is the predicted response.

**Table 3:** Independent Variables and Their Coded Levels Used in the Box–Behnken Design

	levels						
independent variables	symbol	low^a^	middle^a^	high^a^	low^b^	middle^b^	high^b^
pH	*X*_1_	3	4	5	4	6	8
temperature (°C)	*X*_2_	25	40	55	25	40	55
initial dye concentration (ppm)	*X*_3_	50	100	350	100	225	350

a Coded levels for Bent-Ca-Chi.

b Coded levels for Bent-Ca-PEI.

**Table 4:** Box–Behnken Design Matrix

	factors			*R*% of Bent-Ca-Chi		*R*% of Bent-Ca-PEI	
run	*X*_1_	*X*_2_	*X*_3_	experimental	predicted	experimental	predicted
1	–1	–1	0	84.628	85.517	82.276	83.443
2	1	–1	0	76.680	78.108	76.088	76.295
3	–1	1	0	85.744	87.244	85.092	86.630
4	1	1	0	78.772	81.418	78.106	78.683
5	–1	0	–1	75.067	76.727	74.107	74.112
6	1	0	–1	69.722	72.155	67.159	68.124
7	–1	0	1	82.678	83.477	82.118	82.898
8	1	0	1	73.244	74.815	72.052	73.792
9	0	–1	–1	71.249	71.680	67.159	67.161
10	0	1	–1	74.892	76.832	73.132	73.710
11	0	–1	1	77.727	79.019	77.346	78.150
12	0	1	1	76.103	78.904	75.067	77.175
13	0	0	0	98.051	99.731	98.090	99.070
14	0	0	0	98.128	99.731	98.162	99.070
15	0	0	0	98.166	99.731	98.340	99.070

From the BBD results obtained using Minitab, the regression equations with the calculated coefficients for the Bent-Ca-Chi and Bent-Ca-PEI adsorbents are presented in eq 4 and eq 5, respectively:
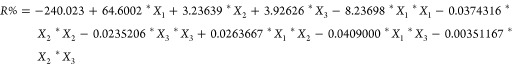
4
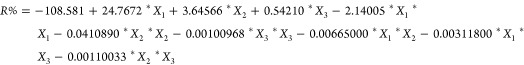
5

The predicted responses for RB-B dye removal by Bent-Ca-Chi and Bent-Ca-PEI adsorbents using eq 4 and eq in 5 were calculated and are presented in Table 4.

The ANOVA results for the quadratic equations are listed in Table 5. The *p*-values of the factors lower than 0.05 suggested that the parameter was significant for dye removal. Table 5 shows that the two-way interaction of *X*_1_∗*X*_2_ was not significant in RB-B adsorption by Bent-Ca-Chi, as the *p*-values were greater than 0.05. The *R*^2^ value of 99.39% indicates a good fit with the model, and the *R*^2^_(adj)_ value of 99.11% demonstrates the significance of the model. Similarly, the dual interactions of *X*_1_∗*X*_2_ and *X*_1_∗*X*_3_ are not significant for RB-B adsorption on Bent-Ca-PEI (*p* > 0.05). The high *R*^2^ value of 98.43% confirmed that the response conforms with the model’s result. Additionally, the lack-of-fit values demonstrated that the second-order model adequately approximated the adsorption data for Bent-Ca-Chi and Bent-Ca-PEI (*p* > 0.05).

**Table 5:** Results of ANOVA for RB-B Removal Percentage by Bent-Ca-Chi and Bent-Ca-PEI Adsorbents

source	DF	adj SS	adj MS	*F* value	*p* value
Bent-Ca-Chi^a^					
model	9	2653.19	294.80	361.29	0.000
*X*_1_	1	220.52	220.52	270.26	0.000
*X*_2_	1	6.83	6.83	8.37	0.009
*X*_3_	1	88.57	88.57	108.55	0.000
*X*_1_^2^	1	501.03	501.03	614.03	0.000
*X*_2_^2^	1	523.803	523.803	641.94	0.000
*X*_3_^2^	1	1595.82	1595.82	1955.73	0.000
*X*_1_·*X*_2_	1	0.48	0.48	0.58	0.454
*X*_1_·*X*_3_	1	8.36	8.36	10.25	0.004
*X*_2_·*X*_3_	1	13.87	13.87	17.00	0.001
error	20	16.32			
lack-of-fit	3	3.32	1.11	1.45	0.264
pure error	17	13.00	0.76		
total	29	2669.51			
Bent-Ca-PEI^b^					
model	9	3101.67	344.63	138.92	0.000
*X*_1_	1	227.83	227.83	91.83	0.000
*X*_2_	1	18.18	18.18	7.33	0.014
*X*_3_	1	156.58	156.58	63.12	0.000
*X*_1_^2^	1	541.37	541.37	218.22	0.000
*X*_2_^2^	1	631.17	631.17	254.41	0.000
*X*_3_^2^	1	1837.96	1837.96	740.86	0.000
X*X*_1_·*X*_2_	1	0.32	0.32	0.13	0.724
*X*_1_·*X*_3_	1	4.86	4.86	1.96	0.177
*X*_2_·*X*_3_	1	34.05	34.05	13.73	0.001
error	20	49.62			
lack-of-fit	3	4.17	1.39	0.52	0.675
pure error	17	45.45	2.67		
total	29	3151.29			

a R^2^ = 99.39%; R^2^(adj) = 99.11%.

b R^2^ = 98.43%; R^2^(adj) = 97.72%.

**Figure 4:**
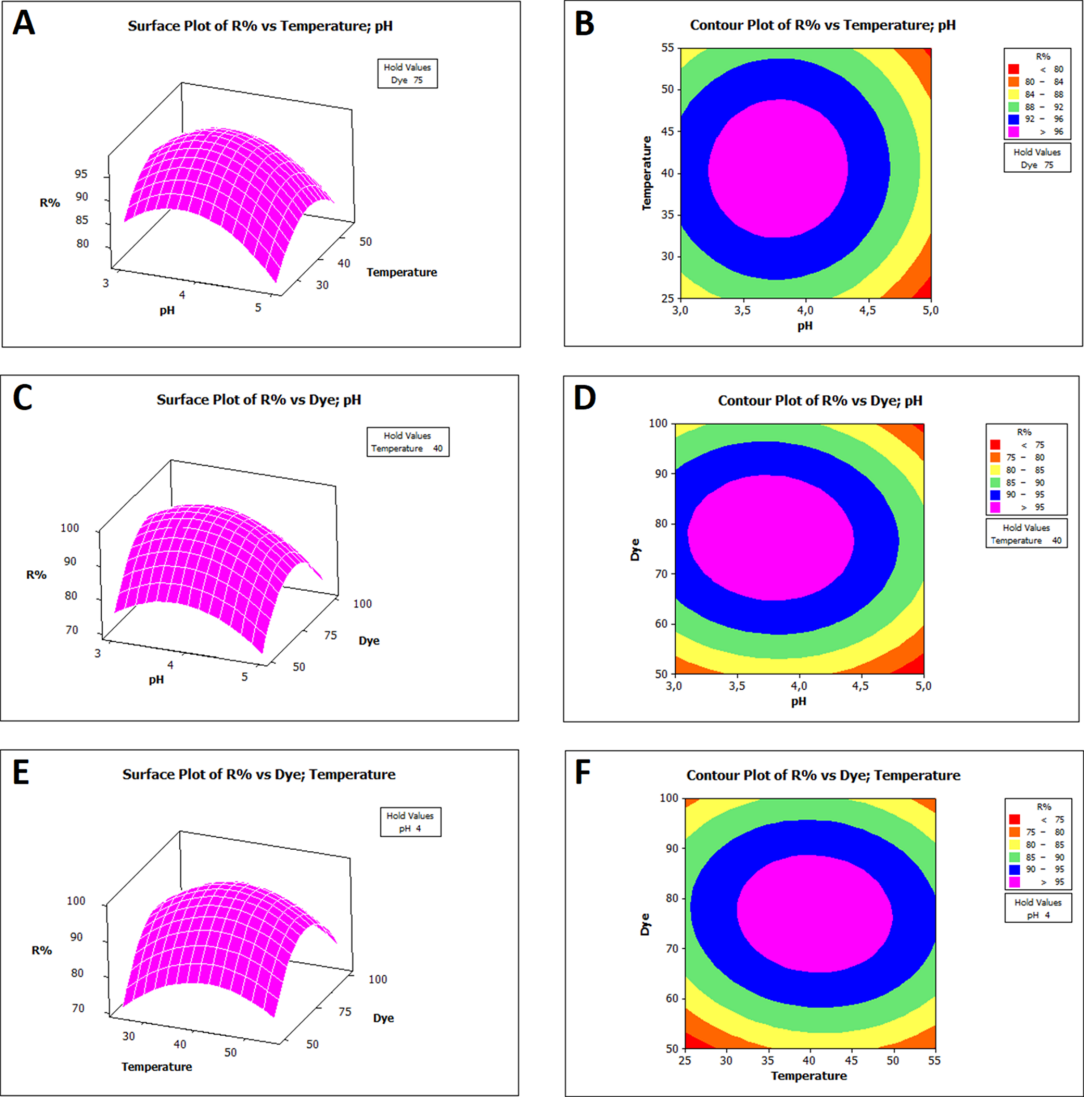
Response surface (A, C, E) and contour (B, D, F) plots showing the effect of pH (*X*_1_), temperature (*X*_2_), and dye (*X*_3_) on the removal percentage of RB-B by Bent-Ca-Chi.

**Figure 5:**
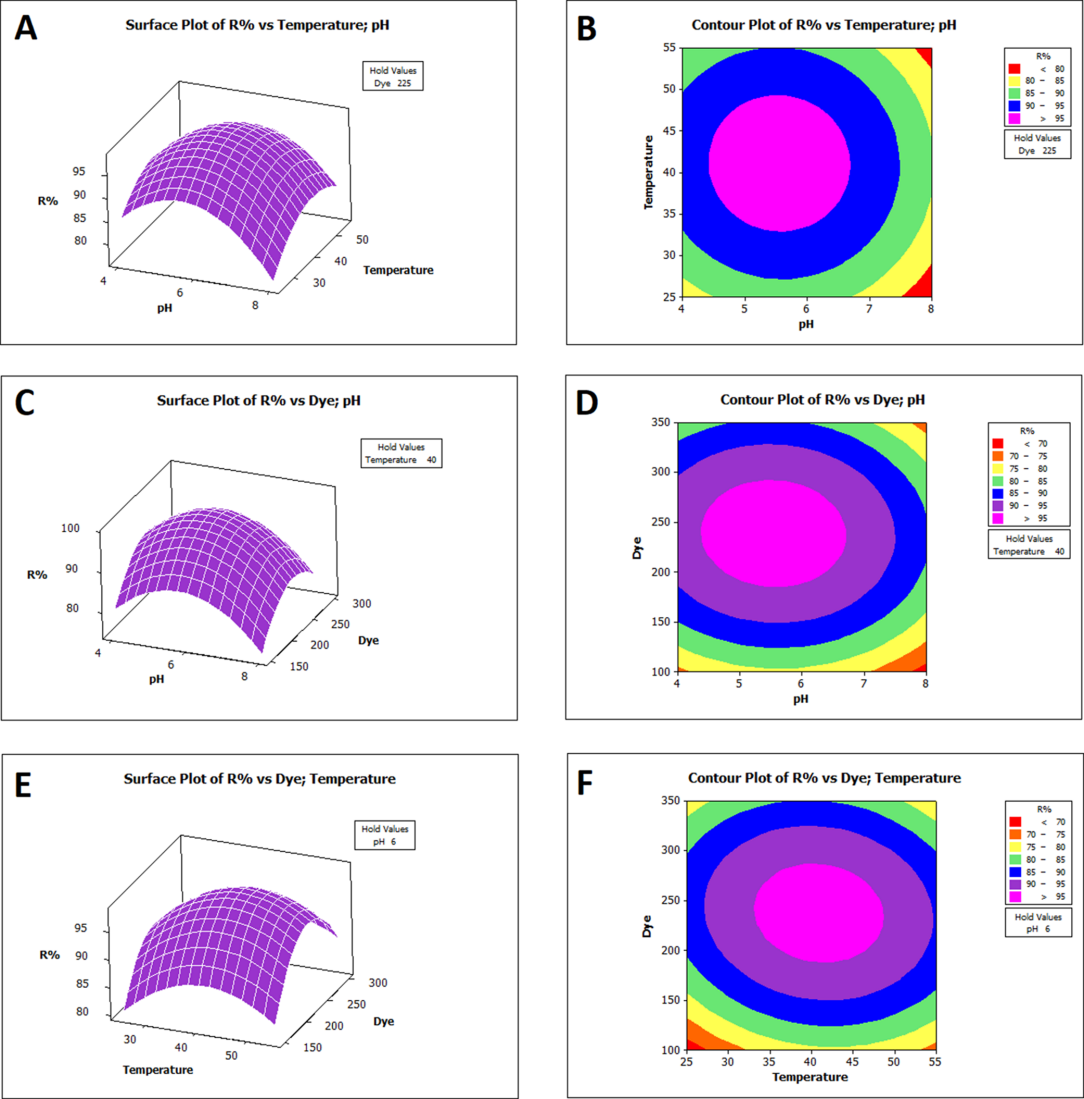
Response surface (A, C, E) and contour (B, D, F) plots showing the effect of pH (*X*_1_), temperature (*X*_2_), and dye (*X*_3_) on the removal percentage of RB-B by Bent-Ca-PEI.

Three-dimensional surface and contour plots based on the RSM are shown in Figures 4 and 5. These plots helped visualize the effects of interactive variables on RB-B adsorption by Bent-Ca-Chi and Bent-Ca-PEI. The spherical surface graphs in the 3D plots indicate that the quadratic equations effectively represent the modeling of RB-B adsorption in RSM optimization.

### 3.4 Optimization of the Response

**Figure 6:**
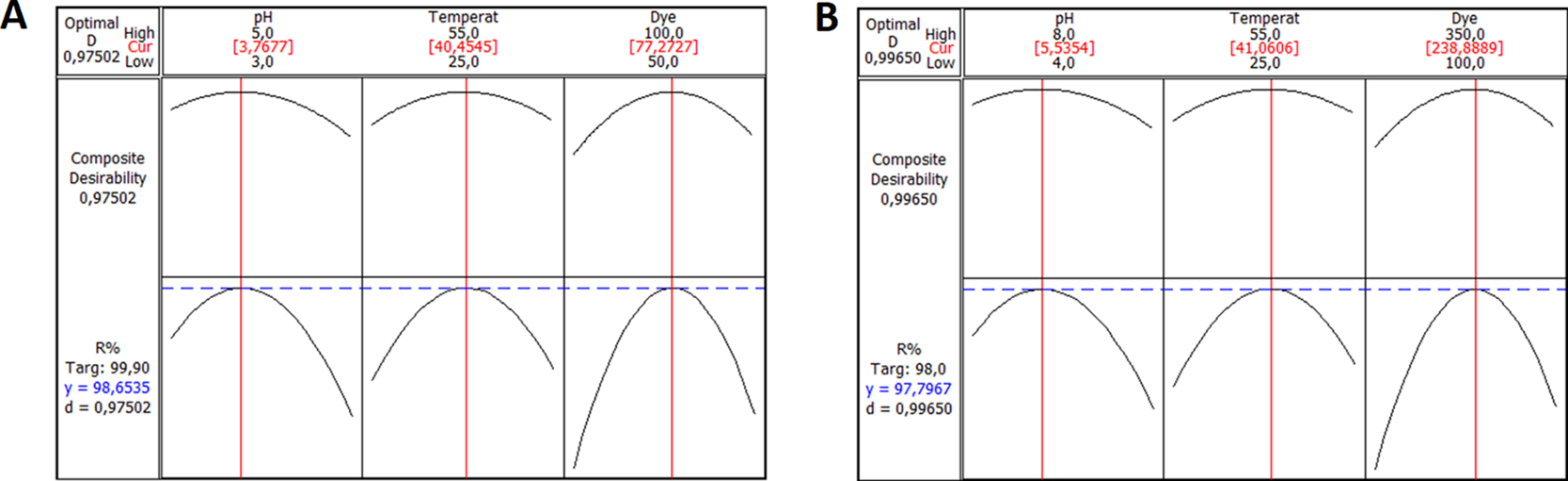
Composite desirability and optimization plots of Bent-Ca-Chi (A) and Bent-Ca-PEI (B).

The optimum conditions for maximum RB-B removal efficiency were determined by using the response optimizer of the Minitab software. Figure 6A,B illustrates the effect of each factor (*X*_1_, *X*_2_, and *X*_3_) of BBD on the response, with the vertical lines indicating the optimum values of the parameters. The dashed horizontal lines represent the actual response value of *Y*, which is the dye removal efficiency, and *d* indicates the desirability, which determines how well the settings optimize or satisfy the desired response. For Bent-Ca-Chi, the optimal combination for maximum dye removal was found with a pH of 3.77, a temperature of 40.45 °C, and a dye concentration of 77.27 mg L^–1^, with an excellent desirability of 0.9750 (Figure 6A). For Bent- Ca-PEI, the predicted optimal values for RB-B removal percentage were a pH of 5.53, a temperature of 41.06 °C, and a dye concentration of 238.89 mg L^–1^, with the desirability of 0.9965 (Figure 6B).

### 3.5 Adsorption Isotherm Models

**Figure 7:**
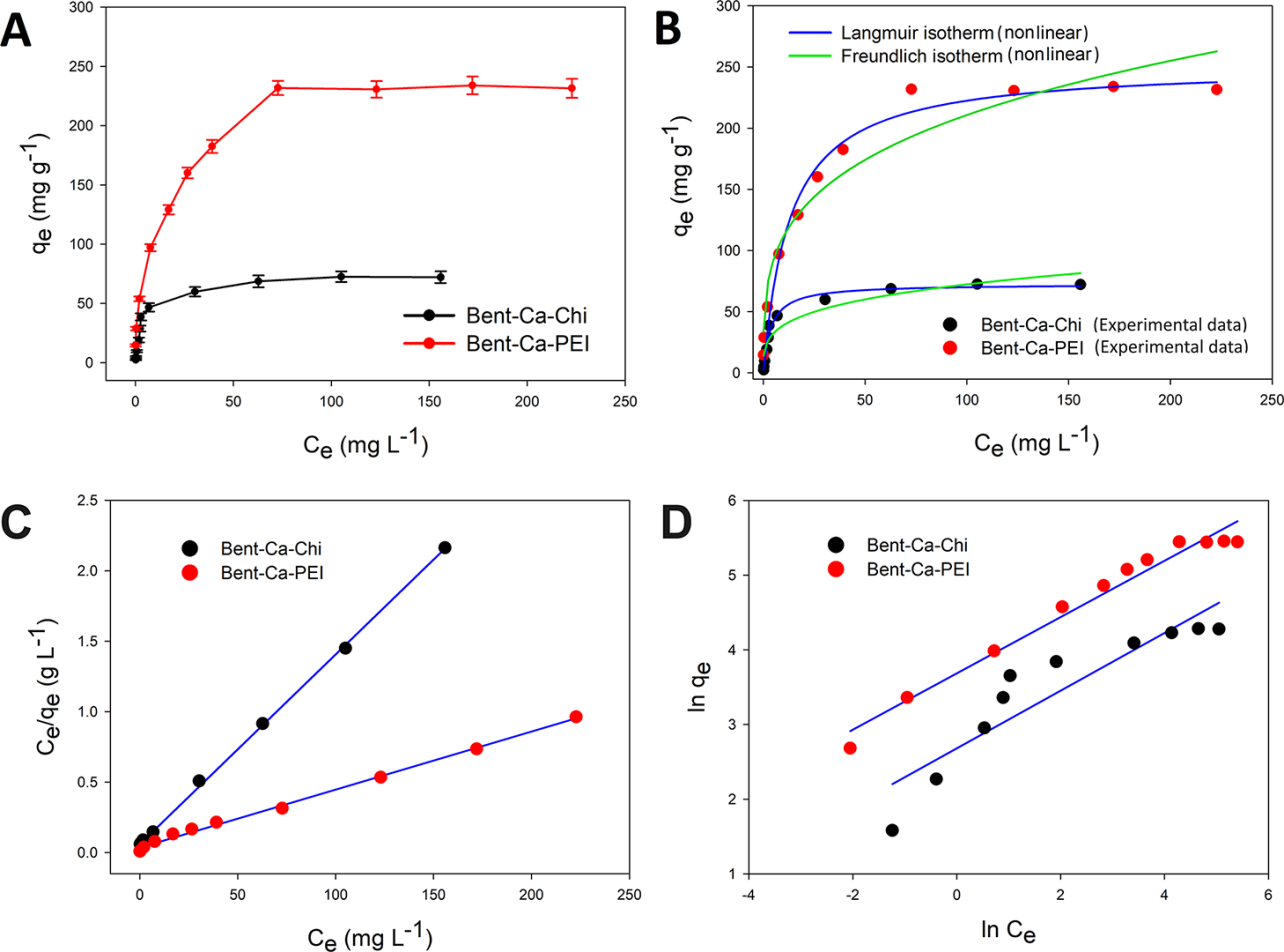
Plot of *q*_e_ vs. *C*_e_ graph (A), nonlinear fitting of Langmuir and Freundlich isotherm models (B), linearized plots of Langmuir (C), and Freundlich (D) isotherm models for RB-B adsorption under the conditions of 0.010 g of the adsorbents, a volume of 5–300 mg L^–1^ dye (pH of 4 and 6, respectively, for Bent-Ca-Chi and Bent-Ca-PEI), 250 rpm of shaking for 4 h, and 40 °C temperature.

Adsorption isotherms are very important for explaining the interaction behavior of the adsorbate and the adsorbent at a given temperature. To describe the adsorption behavior of RB-B on Bent-Ca-Chi and Bent-Ca-PEI, the experimental data were modeled using the well-known adsorption isotherm models of Langmuir and Freundlich. The experimental data was plotted as *q*_e_ vs. *C*_e_ graphs, and nonlinear and linear isotherm models were fitted as shown in Figure 7A–D. Table 6 shows the parameters of the adsorption models calculated from the equations of these isotherm models.

**Table 6:** Isotherm and Kinetic Model Parameters Calculated from Nonlinear and Linear Model Equations for RB-B Adsorption

isotherm model					
		nonlinear form		linear form	
	parameter	Bent-Ca-Chi (*q*_e (exp)_ = 72.06 ± 2.45 mg g^–1^)	Bent-Ca-PEI (*q*_e (exp)_ = 233.92 ± 4.32 mg g^–1^)	Bent-Ca-Chi (*q*_e (exp)_ = 72.06 ± 2.45 mg g^–1^)	Bent-Ca-PEI (*q*_e (exp)_ = 233.92 ± 4.32 mg g^–1^)
Langmuir	*q*_max_ (mg g^–1^)	72.56 ± 1.34	251.48 ± 5.67	74.07 ± 2.41	243.90 ± 5.45
	*K*_L_ (L mg^–1^)	0.2731 ± 0.011	0.0767 ± 0.008	0.2284 ± 0.024	0.1191 ± 0.017
	*R*^2^	0.9844	0.9743	0.9994	0.9965
Freundlich	*K*_F_(mg g^–1^) (L mg^–1^)^1/*n*^	21.04 ± 0.52	59.33 ± 0.96	14.60 ± 0.87	39.84 ± 3.47
	1/*n*	0.2680 ± 0.015	0.2753 ± 0.087	0.3863 ± 0.079	0.3769 ± 0.065
	*R*^2^	0.8848	0.9354	0.8383	0.9717

kinetic model					
		nonlinear form		linear form	
	parameter	Bent-Ca-Chi (*q*_e (exp)_ = 69.73 ± 0.032 mg g^–1^)	Bent-Ca-PEI (*q*_e (exp)_ = 244.53 ± 7.39 mg g^–1^)	Bent-Ca-Chi (*q*_e (exp)_ = 69.73 ± 0.032 mg g^–1^)	Bent-Ca-PEI (*q*_e (exp)_ = 244.53 ± 7.39 mg g^–1^)
pseudo-first-order	*k*_1_ × 10^4^ (min^–1^)	923.2 ± 102.7	2251.5 ± 300.2	29.94 ± 9.77	31.09 ± 1.63
	*q*_e (theo)_(mg g^–1^)	60.08 ± 0.51	218.15 ± 3.24	25.52 ± 3.30	57.35 ± 9.01
	*R*^2^	0.5630	0.4655	0.9466	0.9508
pseudo-second-order	*k*_2_ × 10^4^ (g mg^–1^ min^–1^)	21.90 ± 3.11	17.25 ± 4.17	15.33 ± 3.91	7.03 ± 0.63
	*q*_e (theo)_ (mg g^–1^)	63.15 ± 0.67	225.96 ± 4.62	61.57 ± 1.88	224.75 ± 3.57
	*R*^2^	0.7962	0.7225	0.9988	0.9987
intraparticle diffusion	*k*_p_ (mg g^–1^ min^–1/2^)	0.88 ± 0.04	1.90 ± 0.32	1.30 ± 0.14	2.41 ± 0.38
	*C* (mg g^–1^)	39.77 ± 0.85	179.05 ± 3.72	37.61 ± 1.38	179.41 ± 3.54
	*R*^2^	0.8718	0.8320	0.8755	0.9422

The Langmuir model predicts the monolayer adsorption on the adsorbate surface, which contains many equal energy active sites. Nonlinear and linearized fitting of the Langmuir isotherm model equation can be expressed as in eqs 6 and 7, respectively.
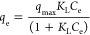
6
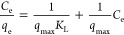
7where *q*_e_ (mg g^–1^) is the amount of dye adsorbed at equilibrium time, *C*_e_ (mg L^–1^) is the equilibrium concentration of the adsorbate, *q*_max_ (mg g^–1^) is the maximum adsorption capacity of the adsorbent, and *K*_L_ (L mg^–1^) is the Langmuir isotherm constant, which is related to the affinity of the binding sites of the adsorbent with the adsorbate. The values of *q*_max_ and *K*_L_ were calculated from the isotherm model equations and are listed in Table 6. High regression coefficients were observed for both nonlinear and linear fits of the models showing that the experimental data fit the Langmuir isotherm model well for the removal of RB-B by Bent-Ca-Chi and Bent-Ca-PEI adsorbents. When a comparison was made between the two types of modeling, it was seen that the isotherm in linear form represented the experimental data very well with significantly higher *R*^2^ values around 0.99. In addition, the nonlinear form of the Langmuir isotherm for RB-B adsorption with acceptable *R*^2^ values shows that the data are consistent with the model. From the isotherm model, the calculated adsorption capacity, which is *q*_max_, was very close to the experimental value (*q*_e (exp)_ (mg g^–1^)) for both adsorbents. From the Langmuir isotherm model, the maximum adsorption capacities of Bent-Ca-Chi and Bent-Ca-PEI were calculated as 74 mg g^–1^ and 244 mg g^–1^, respectively.

The Freundlich isotherm model describes the formation of multilayer adsorption on the heterogeneous surface sides of the adsorbent. The empirical equations for the isotherm model in nonlinear and linear forms are described in eqs 8 and 9, respectively.
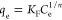
8
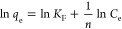
9where 1/*n* and *K*_F_ (mg g^–1^) (L mg^–1^)^1/*n*^ are the Freundlich isotherm parameters obtained from the nonlinear equation of the isotherm model and the slope and intercept of the linearized plot of ln(*q*_e_) vs. ln(*C*_e_), respectively. At equilibrium, *q*_e_ (mg g^–1^) is the adsorbed amount of RB, and *C*_e_ (mg L^–1^) is the concentration of RB. The Freundlich isotherm parameter 1/*n* indicates the favorability of adsorption. When 1/*n* is in the range of 0–1, adsorption is favorable, whereas when it is greater than 1, adsorption becomes invalid, particularly at high concentrations of the adsorbate. Adsorption is linear at low concentrations when 1/*n* = 1. Freundlich isotherm parameters *K*_F_ and 1/*n* were tabulated in Table 6, which shows that RB-B adsorption on the adsorbents is favorable. Furthermore, the regression coefficients (*R*^2^) of the Freundlich isotherm model for the nonlinear and linear forms of the model are lower compared to the Langmuir model, indicating that the Langmuir model better describes the adsorption behavior of RB-B on the adsorbents.

The data of RB-B dye adsorption by cationic charged polymer modified adsorbents in this study have been best fitted to the Langmuir isotherm. The dye adsorption has occurred in a monolayer on active sites of the adsorbent clays, and the maximum adsorption of the dye was the adsorption when the molecules bound to the adsorbent surface form a saturated layer. Moreover, it resulted that the adsorption sites have equal energies with homogeneity and pointed out that there is no interaction between adjacent adsorbed molecules.

The maximum adsorption capacities of Bent-clay-based adsorbents for different anionic dyes are listed in Table 7. If a general comparison is made between some of the Bent clay-based adsorbent capacities given in the table, it is evident that the adsorbents used in this study exhibit high effectiveness in removing anionic charged dyes.

**Table 7:** Comparison of Maximum Adsorption Capacities of Chi or PEI Polymer Modified Bentonite Adsorbents

adsorbent	polymer/bentonite ratio (g/g clay)	dye	*q*_max_ (mg g^–1^)	reference
chitosan/CTAB bentonite	0.2	weak acid scarlet	102.0	31
chitosan/bentonite	1	amido black 10B	323.6	32
chitosan-cobalt oxide/bentonite composite	0.5	congo red	303	34
trimethoxysilane-PEI/bentonite	2.5	amino black 10B	327.7	35
chitosan/bentonite (Bent-Ca-Chi)	0.8	remazol black B	74.0	this study
PEI/bentonite (Bent-Ca-PEI)	0.8	remazol Black B	244	this study

### 3.6 Adsorption Kinetic Models

**Figure 8:**
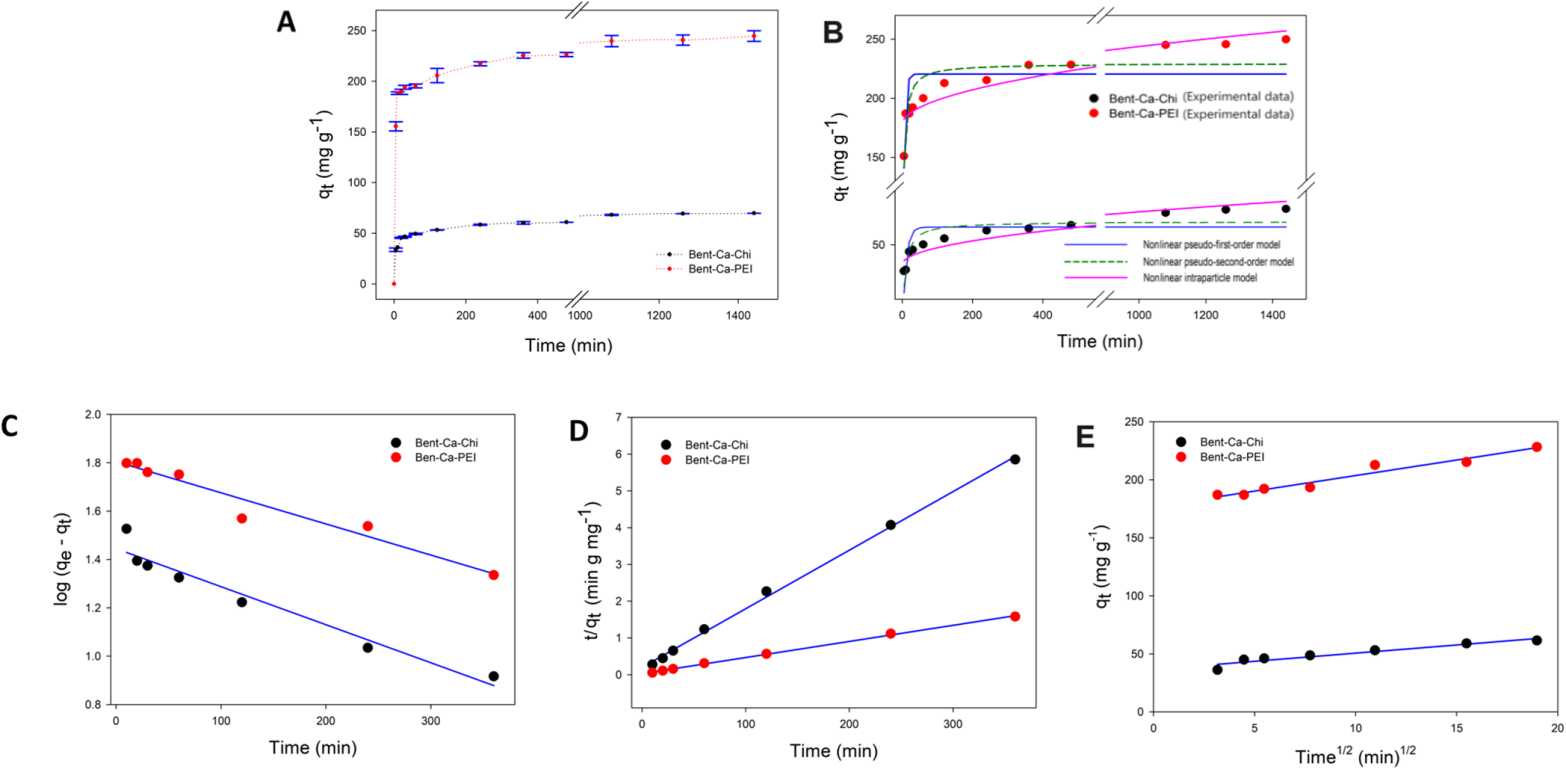
Plot of *q*_t_ vs. time graph (A), nonlinear fitting of pseudo-first-order, and pseudo-second-order, and intraparticle diffusion models (B), linearized plots of pseudo-first-order (C), and pseudo-second-order (D), and intraparticle diffusion (E) kinetic models for RB-B adsorption with 0.010 g of the adsorbents, a volume of 250 mg L^–1^ dye (pH of 4 and 6, respectively, for Bent-Ca-Chi and Bent-Ca-PEI), 250 rpm of shaking for 12 h, and 40 °C temperature.

To investigate the adsorption mechanism and rate-controlling step of RB-B dye adsorption on Bent-Ca-Chi and Bent-Ca-PEI adsorbents, three kinetic models (the pseudo-first-order kinetic model, the pseudo-second-order kinetic model, and the intraparticle diffusion model) were applied to the experimental dye adsorption data. In Figure 8A–E, the graph of *q*_t_ vs. *t* and the nonlinear and linear fitting of kinetic model graphs are shown. The parameters obtained from the adsorption kinetic models are listed in Table 6.

The pseudo-first-order kinetic model assumes that the rate-limiting step of adsorption is physisorption. The nonlinear and linear equations of the model are given in eqs 10 and 11, respectively.
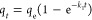
10
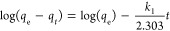
11where *q*_e_ (mg g^–1^) and *q*_*t*_ (mg g^–1^) are the amounts of adsorbate at equilibrium and at time *t*, respectively. *k*_1_ (min^–1^) is the rate constant of the pseudo-first-order kinetic model and can be calculated from linear equation’s slope obtained from the curve of log (*q*_e_ – *q*_t_) vs. *t* (expressed in minutes) graph (Figure 8C). The pseudo-first-order kinetic model in nonlinear and linear form was applied to the kinetic data of RB-B. The rate constants of *k*_*1*_, theoretically calculated *q*_e (theo)_ values, and *R*^2^ are shown in Table 6. The nonlinear form of the pseudo-first-order kinetic model was not found to be suitable to represent the experimental data of RB-B adsorption with very low regression coefficient values. The linear type pseudo-first-order fitted better than the nonlinear form. The *R*^2^ values of the linearized kinetic model for both adsorbents are approximately 0.95. However, the predicted *q*_e_ values did not match well with the experimental values (*q*_e (exp)_ (mg g^–1^)), and the pseudo-first-order kinetic model did not fit the RB-B dye adsorption experimental data.

The pseudo-second-order kinetic model assumes that the rate-limiting step of adsorption is chemisorption. The rate equations of the model in nonlinear and linear forms are given in eqs 12 and 13, respectively.
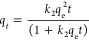
12
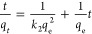
13where *k*_2_ (g mg^–1^ min^–1^) is the rate constant of the pseudo-second-order kinetic model. The rate constant and predicted equilibrium adsorption capacity (*q*_e (theo)_) were calculated from both of the nonlinear curve equation and the slope and intercept values of the linear plot of *t*/*q*_t_ vs. *t* (expressed in minutes) from Figure 8D. The kinetic model parameters for RB-B adsorption on Bent-Ca-Chi and Bent-Ca-PEI are listed in Table 6. The kinetic model showed high regression coefficients (*R*^2^ = 0.99), and the experimental data sets fitted well with the pseudo-second-order kinetic model in linear form, whose *q*_e (theo)_ values were found to be very close compared to the experimental *q*_e (exp)_. Although, the nonlinear pseudo-second-order kinetic model exhibited lower *R*^2^ values, the calculated adsorption capacity from the nonlinear model was compatible with the experimental ones. The well-fitting of linearized pseudo-second-order kinetic model to RB-B adsorption data suggests that between dye molecules and the adsorbent surface, there might have formed strong electrostatic interactions.

The intraparticle diffusion model can be described as in eq 14:
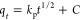
14where *k*_p_ (mg g^–1^ min^–1/2^) is the intraparticle diffusion kinetic model rate constant, and *C* (mg g^–1^) is a constant referring to the boundary layer thickness. The plots of *q*_*t*_ vs. *t*^1/2^ for the adsorbents exhibit a single linear region, as shown in Figure 8E. Thus, it can be assumed that the adsorption mechanism is controlled in a single step. Following the plotting of the nonlinear and linear forms of the model, it was resulted that the intraparticle diffusion model did not match well with the data, and the model is insufficient to represent the adsorption kinetics of RB-B because of low *R*^2^ values.

### 3.7 Reusability of the Adsorbents

**Figure 9:**
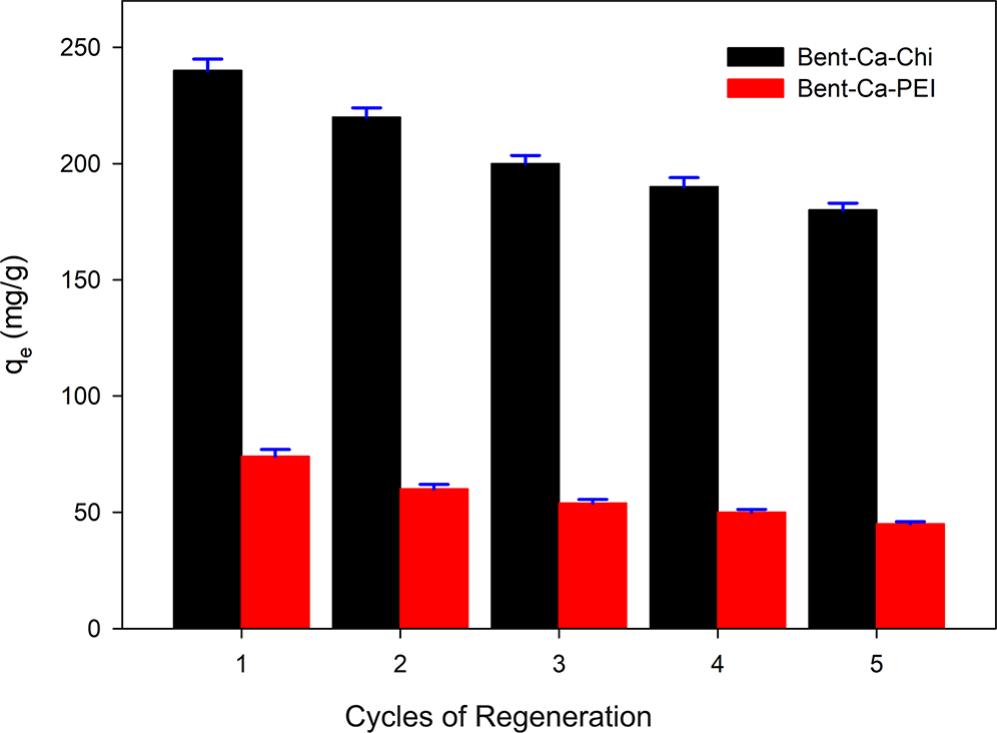
Reusability of Bent-Ca-Chi and Bent-Ca-PEI showing five cycles of regeneration.

The regeneration studies were performed, and the results are given in Figure 9. It was seen that the removal of RB-B dye by Bent-Ca-Chi and Bent-Ca-PEI adsorbents has continued to reach five times usage. The removal efficiency of RB-B was decreased from ∼98 to ∼72% and ∼96 to ∼60% for Bent-Ca-Chi and Bent-Ca-PEI adsorbents, respectively. In the fifth time usage of the adsorbents, the adsorption capacity of Bent-Ca-Chi and Bent-Ca-PEI was found to be 181 mg g^–1^ and 45 mg g^–1^, respectively, for the adsorbents. The decrease in the dye adsorption capability of the adsorbent in terms of dye removal may be associated with deterioration in the active parts of the modified bentonite adsorbents during the treatment of NaOH and washing with distilled water steps. However, the removal efficiency of the adsorbents has shown their sustainability, as they can remove dye at a very high rate even after being renewed and used five times.

## 4 Conclusion

In this study, calcium Bent clay was modified using two polymers, Chi and PEI. The obtained Bent-Ca-Chi and Bent-Ca-PEI clays were subjected to RB-B dye adsorption, and their adsorption capacities were compared. FFD was used to select the effective factors influencing RB-B adsorption. Based on the FFD results, three factors (pH, temperature, and initial dye concentration) significantly affected the adsorption. A three-level BBD combined with an RSM was used to evaluate and optimize adsorption. Isotherm and kinetic modeling were applied to the kinetic data of dye adsorption under the optimum conditions predicted by RSM. The Langmuir isotherm and pseudo-second-order kinetics models were found to accurately describe RB-B adsorption on both clays. It was observed that Bent-Ca-PEI interacted more with the anionically charged dye than Bent-Ca-Chi. It was concluded that composite clay samples, similar to the RB-B structure, are recommended as low-cost and nontoxic materials for the removal of textile dyes in industrial applications.
